# Editorial: Gendered Paths into STEM. Disparities Between Females and Males in STEM Over the Life-Span

**DOI:** 10.3389/fpsyg.2019.02758

**Published:** 2019-12-17

**Authors:** Bernhard Ertl, Silke Luttenberger, Rebecca Lazarides, M. Gail Jones, Manuela Paechter

**Affiliations:** ^1^Institute of Education, Universität der Bundeswehr München, Neubiberg, Germany; ^2^Institute for Early Childhood and Primary Teacher Education, University College of Teacher Education Styria, Graz, Austria; ^3^Department of Education, University of Potsdam, Potsdam, Germany; ^4^Department of STEM Education, North Carolina State University, Raleigh, NC, United States; ^5^Department of Psychology, University of Graz, Graz, Austria

**Keywords:** attributions, self-concept, motivation, gender-sensitive didactics, occupational choices

Choosing a career path into STEM (Science, Technology, Engineering, and Mathematics) is a longitudinal process rather than an *ad-hoc* decision: experiences in childhood and school form individuals' interests, motivation, and ability beliefs—their expectations according to Eccles et al. (1983). These serve as basis for a decision against or toward STEM. However, while youth are considering careers, barriers can emerge, for example students may form stereotyped impressions of STEM as a “male” domain or develop perceptions that brilliance is a prerequisite for STEM attainments. Such assumptions downgrade expectations and often shape women's as well as minority students' self-evaluation of not being suited to a career in STEM.

Altogether, deciding for and following a specific career path is a developmental process (Gottfredson, 2005) of circumscription and compromise and female students often rule out STEM professions during this process. According to expectancy-value theories (EVT; e.g., Eccles et al., 1983), an individual evaluates during this process the balance between the personal expectations for success (resp. activity specific ability beliefs) and the subjective task value for achievement-related values, engagement, and persistence. This evaluation is influenced by the broader context of socializers and the milieu that frame the individual's perceptions and interpretations of experiences. Many papers in this Research Topic refer to this theoretical approach.

While EVT focus the interactions of the different factors during balancing expectancies and values, the Social Cognitive Career Theory (SCCT; Lent et al., 1994, 2018) proposes a step-wise model how personal and environmental variables interact to finally shape choices for performance domains and attainment. The model proposes that (1) person inputs (like predispositions and gender) as well as (2) background contextual affordances and societal characteristics (like cultural norms) shape (3) learning experiences that lead to individual attainments (10) which then may receive feedback from the environment. These learning experiences contextualize an individual's expectations regarding one's self-efficacy (4) and consequently also one's expectations about outcomes of one's actions and attainments (5). Task values such as utility values or interest (7) develop out of self-efficacy and outcome expectations and provide a basis for choice goals (8) and choice actions (9). However, contextual influences proximal to choice behavior (6) also influence interests and choices. Finally, (10) performance domains and attainments result from choice actions.

When connecting the model of the SCCT with Gottfredson's (2005) assumptions of developmental processes, it becomes clear that choice actions do not simply result from predispositions, aptitudes, learning experiences, self-assessments, and interests but that contextual factors moderate such processes. While EVT describe the complex interactions of this moderation process (see Eccles et al., 1983), SCCT rather focuses the steps from the individual's personal inputs toward choice actions moderated by expectancies and contextual factors. These are especially important for female students' career paths into STEM because cultural stereotypes of STEM as a “male” domain as well as interactions with teachers and significant others may influence women to steer away from STEM to more “female” domains or to not consider a STEM career at all (see Ertl et al., 2017).

Against this background, the present Research Topic investigates career decisions, to illustrate the complexity and difficulties of steering more females onto a STEM career path, as well as to summarize evidence about female students' career paths into STEM. The Research Topic comprises 30 articles by 94 authors from ten countries in Europe, America, Oceania, Asia, and Africa. We will structure the editorial according to the factors of SCCT that include expectancies as well as the steps toward a decision for STEM.

(1) **Person Inputs**
  Person inputs may affect self-assessments, motivation, behavior, and thus attainments and can be seen as a starting point for a career path into STEM. If, for example, a person finds his or her aptitude in STEM lacking and conceives her or his talents being outside of STEM, she/he is hardly likely to go into a STEM field. Person inputs are investigated with respect to motivational, emotional, cognitive, or socio-demographic aspects. In the studies presented in this Research Topic motivational aspects relate to goal orientations (Wolter et al.), emotional aspects to empathy as predictor for math achievement (Ghazy et al.), and cognitive aspects relate to visuospatial skills that are often seen as key aptitude for STEM with a clear gender difference in favor of men. Abad et al. as well as Sanchis-Segura et al. look deeper into the issue of visuospatial skills raising the point that tests for the respective skills are often subject to gender framing, a background context, that affects their results. Such background contexts can be also found in Hsieh et al.'s work that focuses on ethnicity and immigrant status as predictors for motivational beliefs.
(2) **Background Contextual Affordances**
  Background contextual affordances relate to culture and cultural norms in which a person is embedded. They provide an indirect impact on all contributions of this Research Topic. The impact of this factor becomes most obvious in the contributions by Sachnis-Segura et al., who discuss how tests for visuospatial performance are constructed (favoring predominantly men), and by Hsieh et al. and Watson et al. who both discuss differences between ethnicities that may include different cultural values.
(3) **Learning Experiences**
  Learning experiences play a major role in the model of Lent et al. (1994) by shaping a student's self-efficacy and outcome expectations. They shape a students' feeling of belonging to a learning domain or not (Banchefsky et al.; Deiglmayr et al.; Höhne and Zander). In a long-term process, learning experiences are related to attainments, outcomes, and subsequent feedback and thus mostly need a longitudinal design for their investigation. In the Research Topic, several longitudinal studies are concerned with these variables, especially Dietrich and Lazarides and Hsieh et al. who investigate the development of motivational belief patterns, and Vinni-Laakso et al. as well as Watson et al. who focus on the long-term development of students' self-concept.
(4) **Self-Efficacy and Self-Concept Expectations**
  Self-efficacy expectations, for example, are subject-specific academic self-concept or ability beliefs. Self-efficacy expectations are a crucial aspect of career paths into STEM and often vary by gender. Large scale studies such as PISA (OECD, 2015) confirm that—even in case of identical academic outcomes and assessments—the self-concept for STEM is lower for female than for male students. Consequently, several contributions delve deeper into self-concept and ability beliefs. For example, Watson et al. looks closer into the gender-related decline of the self-concept in mathematics. Factors contributing to such processes and to the development of a student's self-concept for STEM in general are investigated by Heyder et al. who explore the impact of teacher expectations as well as by Höhne and Zander who analyze the impact of belongingness. The impact of the self-concept on further developments is investigated by Han who analyzes the relationship between self-concept and achievements, by Luttenberger et al. as well as by Sobieraj and Krämer who focus on the relationship between self-concept and motivation in STEM, and by Saß and Kampa who investigate the impact of self-concept profiles on course selection. Finally, Dietrich and Lazarides as well as Vinni-Laakso et al. analyze to which degree motivational belief patterns are associated with math-related career plans.
(5) **Expectations About Outcomes**
  While self-efficacy expectations focus on the estimation of one's own ability, outcome expectations result from an assessment to which degree one's own skills are sufficient to achieve satisfactory outcomes in a field. In this sense, Kessels (2015) discusses women's belief that success in STEM careers is based on innate talent or even brilliance (which women typically believe not to have in STEM) as opposed to hard work and diligence. Consequently, female students often shy away from STEM career choices even if they achieve good grades. Such field-specific ability beliefs, for example that a successful STEM career requires brilliance, have impacts on women's emotions and motivation in STEM fields. Therefore, Deiglmayr et al. as well as Höhne and Zander investigate in a sample of female students and the degree to which such beliefs are associated with uncertainty and feelings of not belonging to the domain of STEM even though these students major in a STEM field. Luttenberger et al. investigate the degree to which such beliefs predict motivation in STEM and Lazarides and Lauermann explore how beliefs may affect students' career plans. Hsieh et al. as well as Dicke et al. analyze how such beliefs develop over a long range in different STEM subjects and finally Bailey et al. investigate STEM and non-STEM undergraduates as well as academics discussing to which degree undergraduates' beliefs about talent in academia mirror those of academics.
(6) **Contextual Influences Proximal to Choice Behavior**
  Thereby, Bailey et al. investigate contextual influences exerted by others and take up the hypothesis, that university graduates transfer their (stereotypical) ability beliefs to undergraduates. Such phenomena are in the scope of several contributions of this Research Topic focusing on the influence of teachers (Heyder et al.), parents (Hoferichter and Raufelder; Luttenberger et al.; Schorr), or the peer-group (Sáinz et al.). Apart from these personal influences, STEM subjects are often generally attributed stereotypically as being male, an aspect that is taken up by Makarova et al. as well as by Watson et al., and consequently female students in STEM often choose contexts that are to a lower degree regarded as being typically male subjects, for example biology instead of physics contexts if they are able to choose (see Wheeler and Blanchard). Such stereotypical perspectives may be reinforced by representations in TV, which is investigated by Wille et al. Generally, such stereotypical as well as traditional gender role beliefs taken up from personal contexts predict lower educational attainment and less inclination for studying STEM subjects—an issue that is investigated by Dicke et al.
(7) **Interests and Task Values**
  Interests develop and deepen partly due to an individual's self-efficacy and outcome expectations—however, they are also shaped by contextual influences, for example, when interests are regarded as being inappropriate for a specific gender or when pursuing them seems to require too much effort (see for example Gottfredson, 2005) or task values (see Eccles et al., 1983). In this line, Song et al. investigate the impact of interest and effort on persistence. However, as Schorr discusses, interest is often subject to pre-conditions including personal competency and outcome expectations. Similarly, Sobieraj and Krämer analyze to which degree self-perceptions are conjoined with interest-related characteristics such as intrinsic motivation. In this sense, Ertl and Hartmann as well as Watt et al. bridge the gap between interest and motivational profiles and respective choice goals and actions. Lazarides and Lauermann investigated this relation with respect to task values and career aspirations.
(8) **Choice Goals**
  Choice goals can be defined as students' career aspirations that either can go along with a student's interests or reveal deviations. Here, Ertl and Hartmann analyze to which degree students' interests fit to their career aspirations and they find a worse fit between interest and aspirations for STEM than for other subjects. Watt et al. identify different motivational profiles and discuss that especially disengaged students show lower STEM aspirations. Motivation and motivational belief-patterns and their impact on career plans are also discussed by Dietrich and Lazarides as well as by Lazarides and Lauermann, while Vinni-Laakso et al. analyze the impact of self-concept profiles on science course selection. Makarova et al., finally, expand the view on choice-goal section by discussing the impact of gender-science stereotypes on students' choice goals.
(9) **Choice Actions**
  Specific choice actions are less predominant in the Research Topic, possibly because the transformation from a choice goal to a choice action is difficult to observe and to operationalize. Despite of such difficulties, Saß and Kampa aim at explaining science course selection by the impact of self-concept profiles. Sobieraj and Krämer apply a retrospective approach for explaining differences between STEM and non-STEM master students with respect to competence, motivational, and volitional variables.
(10) **Performance Domains and Attainment**
  Ideally, those who have embarked on a STEM career and show persistence should experience satisfying outcomes such as high attainments (grades, professional success), feelings of belonging, joy, or life satisfaction. The two contributions which look closer into these concepts show that students' persistence in STEM is related to a feeling of belonging (Banchefsky et al.) and to interest (Song et al.). Regarding outcomes, Ghazy et al. analyze the role of empathy for math achievement and math scores and find different effects for male and for female students. Han also focuses on math performance scores and find a stereotype effect impeding female pupils. Similarly, Wille et al. investigate STEM stereotypes in a learning context and their differential effects on scores, stereotype endorsement, and belongingness. Sanchis-Segura et al. find similar effects for visuospatial tasks. The effect of framing tasks differently is investigated by Wheeler and Blanchard focusing on how biological contexts may facilitate female students' familiarizing with the context of force rather than traditional physics contexts. Hoferichter and Raufelder investigate the impact of parents' support and pressure on STEM performance and disclose differential effects for female and male students.

## Development of STEM Pathways

A specific aim of this Research Topic is to shed light on critical incidents or milestones on a STEM pathway over the life-span. Therefore, the topic covers evidence from kindergarten (Abad et al.) to adult STEM professionals (Dicke et al.). In between, all stages of formal education are well-covered including primary school (Han; Heyder at al.; Vinni-Laakso et al.; Watson et al.), lower secondary school (Hoferichter and Raufelder; Saß and Kampa; Song et al.; Wille et al.), and upper secondary school (Dietrich and Lazarides; Hsieh et al.; Lazarides and Lauermann; Makarova et al.; Schorr; Watt et al.; Wheeler and Blanchard) with a clear focus on university education (Bailey et al.; Banchefsky et al.; Deiglmayr et al.; Ertl and Hartmann; Ghazy et al.; Höhne and Zander; Luttenberger et al.; Sáinz et al.; Sanchis-Segura et al.; Sobieraj and Krämer; Wolter et al.). Some of these contributions focus on longitudinal developments, for example Abad et al. on the development of visuospatial skills, Dietrich and Lazarides as well as Hsieh et al. on the development of motivational belief patterns, Hoferichter and Raufelder on grades and parental influences, Vinni-Laakso et al. and Watson et al. on students' self-concept, and Dicke et al. on the development of STEM professionals.

## Heterogeneity of STEM Subjects

Although the term STEM raises the impression of being a homogeneous academic domain, there are different definitions which vary in their broadness and some of them even include life sciences and social sciences into STEM (for a discussion, see Ertl et al., 2017). This Research Topic focuses on the core of STEM that covers natural sciences, technology, engineering, and mathematics. However, also within this narrow definition of STEM, authors point at differences between the subjects. They can be distinguished with respect to the proportion of women in a field (Ertl and Hartmann; Luttenberger et al.), with respect to specific subjects as for example in comparisons of mathematics and biology (Hoferichter and Raufelder); research can also refer to science in general (Watt et al.) or to a range of different subjects in the field of STEM (Deiglmayr et al., Hsieh et al.; Makarova et al.). Contributions that focus on one subject mostly investigate mathematics as key subject in STEM (Dietrich and Lazarides; Ghazy et al.; Han; Heyder et al.; Song et al.; Watson et al.; Wille et al.), followed by computer science (Höhne and Zander; Schorr) and physics (Wheeler and Blanchard). To uncover the special characteristics of STEM, some authors compare STEM subjects with NON-STEM (Bailey et al.; Dicke et al.; Ertl and Hartmann; Lazarides and Lauermann; Sanchis-Segura et al.; Sobieraj and Krämer; Wolter et al.). The remaining contributions focus on rather general aspects regarding STEM (Abad et al.; Banchefsky et al.; Luttenberger, Steinlechner et al.; Sáinz et al.; Saß and Kampa, as well as Vinni-Laakso et al.).

## Facilitation Gendered Pathways into STEM

Luttenberger, Steinlechner et al. finally comment on the development on an individual's STEM pathway from interests to a career goal and choice actions and its respective facilitation by shedding light on the importance of early career-related learning experiences as well as on removing external barriers on the path into STEM.

## Author Contributions

BE, SL, RL, MJ, and MP wrote this editorial.

### Conflict of Interest

The authors declare that the research was conducted in the absence of any commercial or financial relationships that could be construed as a potential conflict of interest.
